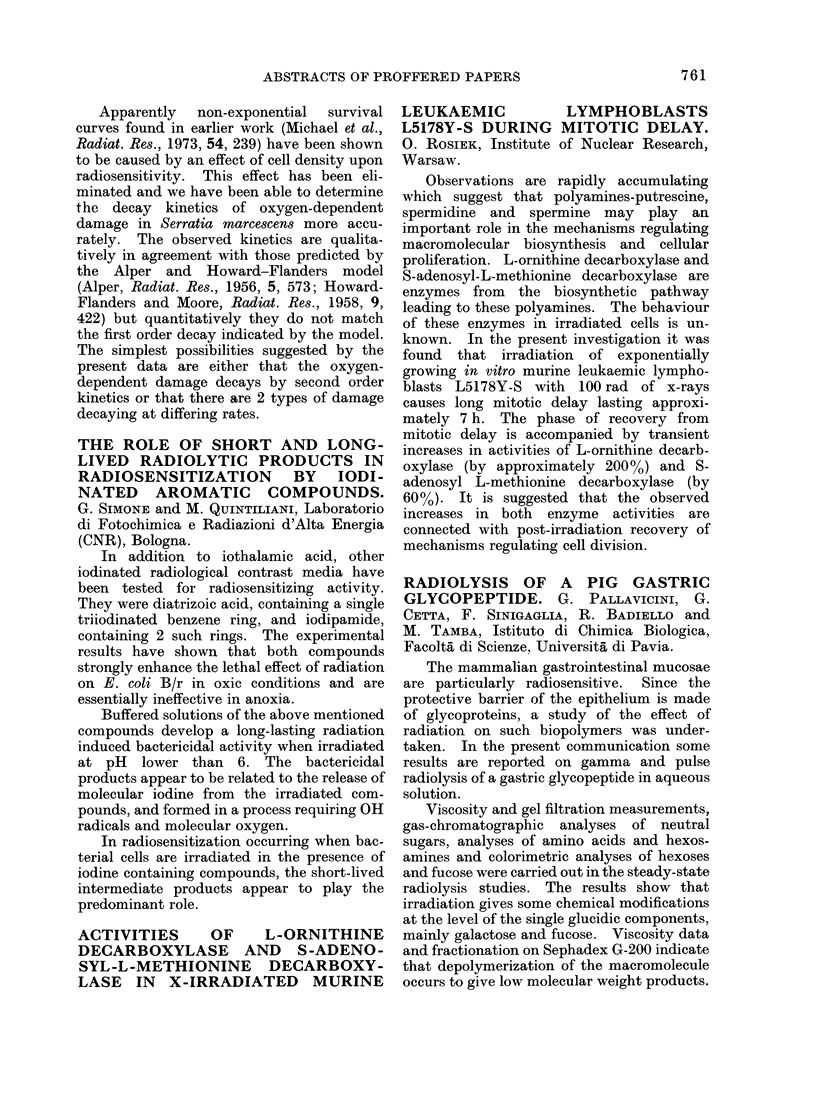# Proceedings: Activities of L-ornithine decarboxylase and S-adenosyl-L-methionine decarboxylase in x-irradiated murine leukaemic lymphoblasts L5178Y-S during mitotic delay.

**DOI:** 10.1038/bjc.1975.323

**Published:** 1975-12

**Authors:** O. Rosiek


**ACTIVITIES OF L-ORNITHINE DECARBOXYLASE AND S-ADENOSYL-L-METHIONINE DECARBOXYLASE IN X-IRRADIATED MURINE LEUKAEMIC LYMPHOBLASTS L5178Y-S DURING MITOTIC DELAY.** O. ROSIEK, Institute of Nuclear Research, Warsaw.

Observations are rapidly accumulating which suggest that polyamines-putrescine, spermidine and spermine may play an important role in the mechanisms regulating macromolecular biosynthesis and cellular proliferation. L-ornithine decarboxylase and S-adenosyl-L-methionine decarboxylase are enzymes from the biosynthetic pathway leading to these polyamines. The behaviour of these enzymes in irradiated cells is unknown. In the present investigation it was found that irradiation of exponentially growing *in vitro* murine leukaemic lymphoblasts L5178Y-S with 100 rad of x-rays causes long mitotic delay lasting approximately 7 h. The phase of recovery from mitotic delay is accompanied by transient increases in activities of L-ornithine decarboxylase (by approximately 200%) and S-adenosyl L-methionine decarboxylase (by 60%). It is suggested that the observed increases in both enzyme activities are connected with post-irradiation recovery of mechanisms regulating cell division.